# Advancing the evidence to improve the nutrition of populations: a refreshed vision and scope for *Nutrition Journal*

**DOI:** 10.1186/s12937-017-0263-4

**Published:** 2017-07-26

**Authors:** Sharon I. Kirkpatrick, Clare E. Collins

**Affiliations:** 1 School of Public Health and Health Systems, University of Waterloo, Waterloo, ON Canada; 2 School of Health Sciences and Priority Research Centre in Physical Activity and Nutrition, University of Newcastle, Callaghan, NSW Australia

## Background

This editorial introduces a revised and refreshed scope for *Nutrition Journal*, one of BioMed Central’s open access journals. Diet is among the leading risk factors for morbidity and mortality globally [1]. This signals the need for high-quality policy-relevant nutrition research to advance the evidence base on strategies to promote and support healthy eating, as well as effective dissemination of that research.

## Scope

With its refreshed scope, *Nutrition Journal* will prioritize manuscripts reporting on novel and high-impact research shedding light on factors that influence eating patterns among diverse populations, and studies that elucidate relationships between eating patterns and health and disease outcomes. We also welcome intervention research, including controlled trials but also natural experiments that uncover successes and challenges in implementing strategies to improve eating patterns among populations, including interactions between policies and programs and contextual factors. Within these areas, we invite manuscripts reporting on research conducted with various populations across the lifecycle and in diverse settings. Considered is research within public and population health, as well as in clinical settings and among individuals with chronic disease risk factors or endpoints.

Dietary assessment is fundamental to much nutrition research. With the refreshed scope, the journal places a more explicit focus on methodological considerations. We encourage authors to peruse publicly-available resources [2–4] related to the selection of the most appropriate methods and measures for assessing dietary intake within a particular study, an area that remains a challenge. We welcome manuscripts that advance methods for assessing dietary intake and food-related behaviours, a lively area of research. Consideration is open to papers reporting on promising methods and tools for capturing dietary exposures and outcomes that incorporate web-based and mobile technology, biomarkers and metabolomics, and other novel approaches such as spectroscopy. We will also consider papers offering insights into strategies to mitigate error in dietary intake data, including statistical advances framed for dissemination to our broad readership.

In all papers reporting on studies including the measurement of dietary intake, we encourage perusal of the checklist associated with the nutritional epidemiology extension of the Strengthening the Reporting of Observational Studies in Epidemiology (STROBE) Statement, known as STROBE-Nut [5]. Although targeted to nutritional epidemiology, considerations listed in the STROBE-Nut checklist related to detailed reporting of methods of dietary assessment are also relevant to surveillance and intervention research. The EURopean micronutrient RECommendations Aligned (EURRECA) Network of Excellence scoring system to assess dietary intake validation studies is also a useful resource in considering study quality [6]. More broadly, the journal requires the use of relevant Enhancing the QUAlity and Transparency Of health Research (EQUATOR) guidelines [7], such as the CONSORT statement [8] for clinical trials and SRQR [9] for qualitative research, to enhance reporting quality.

Study protocol manuscripts will be considered in light of the aim of increasing transparency of methodology such that findings can be interpreted appropriately. Publication of protocols is also intended to contribute to the harmonization of methods across studies so the resulting evidence can be synthesized in a meaningful way. The journal thus welcomes the submission of manuscripts describing protocols for cohort studies and controlled trials, including studies designed to provide baseline data to enable leveraging natural experiments (e.g., policies related to food availability) to offer novel insights. As above, within such papers, we seek detailed descriptions of the dietary methods and measures used to assess dietary exposures and outcomes and the considerations weighed in arriving at these methods and measures, as well as the planned use of statistical approaches to mitigate error in dietary data. Protocols for randomized controlled trials should be accompanied by the checklist corresponding to the SPIRIT Statement [10, 11].

The journal will continue to consider systematic reviews and meta-analyses that adopt robust methods for the identification of relevant literature and abstraction of results. Authors of such papers should consult the Preferred Reporting Items for Systematic Reviews and Meta-Analyses (PRISMA) [12] statement early on in the planning process and make use of the PRISMA checklist in the development of manuscripts.

Finally, given that publication in the peer-reviewed literature is only one of many approaches to dissemination with reach limited to particular audiences, the journal also encourages the submission of papers advancing the science of knowledge translation in the area of nutrition evidence, both to health professionals and members of the public.

With our refreshed scope, we are embracing a theme of holism. The journal has a strong tradition of publishing research reporting on the impact of specific dietary constituents on risk factors for disease. We will continue to consider such studies provided a clear rationale is articulated, but encourage researchers to consider eating patterns more holistically given increasing recognition that it is the combination of foods and beverages we consume rather than particular nutrients or other dietary components in isolation that influence our health [13]. Thus, we invite papers that advance methods for understanding eating patterns and diet quality and their influence on health over the lifecycle. Similarly, in terms of intervention studies, to address gaps in the literature, we will prioritize manuscripts that take a holistic or systems perspective [14] in terms of considering the various factors that can promote or hinder the success of different strategies—these factors include contextual influences [15] that might determine whether an intervention shown to be effective in one setting will perform similarly in another. Intervention studies should also consider the potential for trade-off or compensatory effects among dietary components, as well as for differential effects among varied subgroups within a population. These include subgroups differentiated by sex and gender [16].

Given our focus on advancing our understanding of nutrition and its influences in populations, the journal does not publish animal studies.

## About the journal

*Nutrition Journal* is an open access, broad scope journal that focuses on human and population nutrition. All articles published by Nutrition Journal are made freely and permanently accessible online immediately upon publication, without subscription charges or registration barriers. Nutrition Journal operates an open peer-review system, which increases transparency and accountability through the identification of reviewers. Additional details regarding article types and submission guidelines can be found at https://nutritionj.biomedcentral.com/.

## Conclusion

We are very pleased to invite you to submit your research manuscripts to *Nutrition Journal*. Our vision is to create a journal recognized for publishing high-quality human nutrition research with a focus on transparency and robustness of methods, particularly those used for measuring dietary exposures and outcomes and related dietary phenomena.
